# Supplementary material for: Mitochondrial prohibitin complex regulates fungal virulence via ATG24-assisted mitophagy
Source: Commun Biol. 2022 Jul 14;5:698. doi: 10.1038/s42003-022-03666-5 (PMC9283515; doi:10.1038/s42003-022-03666-5)
Supplement: Supplementary file 2 — Supplementary Information [file 42003_2022_3666_MOESM2_ESM.pdf]

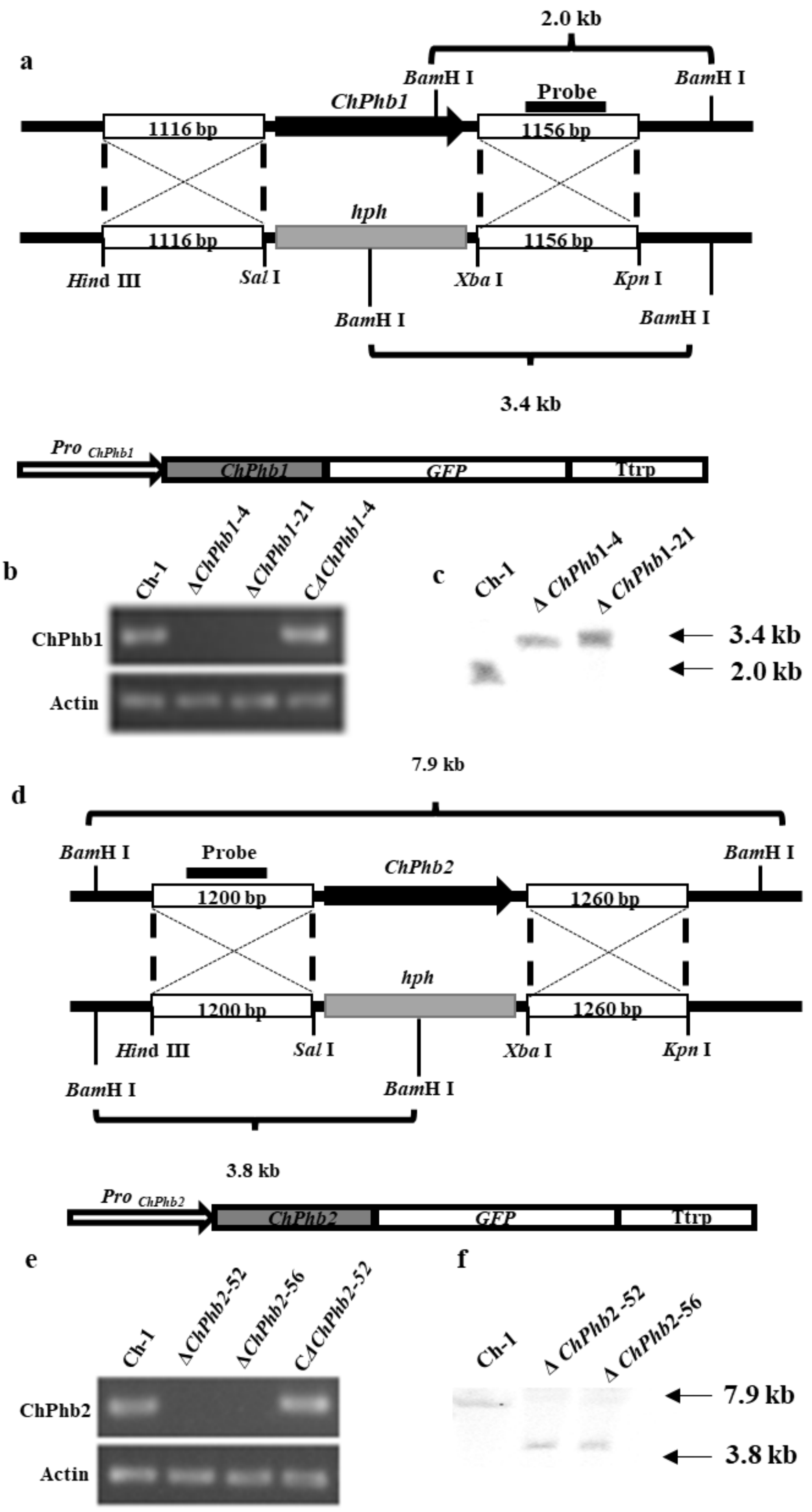

Supplementary Fig. 1. Characterization, targeted deletion, and complementation of *ChPhb1* and *ChPhb2*. **a**, Schematic map of the *ChPhb1* deletion and complementation constructs and restriction enzyme sites. **b**, RT-PCR analysis of the *ChPhb1* deletion and complementation mutants. **c**, Southern blot confirmation of the *ChPhb1* mutants. **d**, Strategic map of the *ChPhb*2 deletion and complementation constructs and restriction enzyme sites. **e**, RT-PCR analysis of the *ChPhb2* deletion and complementation mutants. **f**, Southern blot confirmation of the *ChPhb2* mutants.

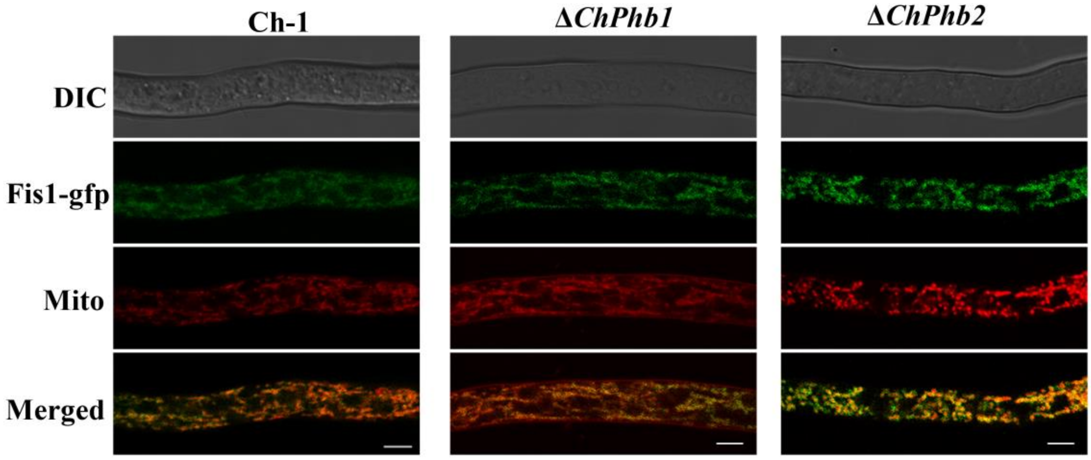

Supplementary Fig. 2. ChPhb1 and ChPhb2 are not essential for mitochondrial fission. Mitochondria in hyphae of the strains were observed under a confocal fluorescence microscope. Scale bar, 5 μm.

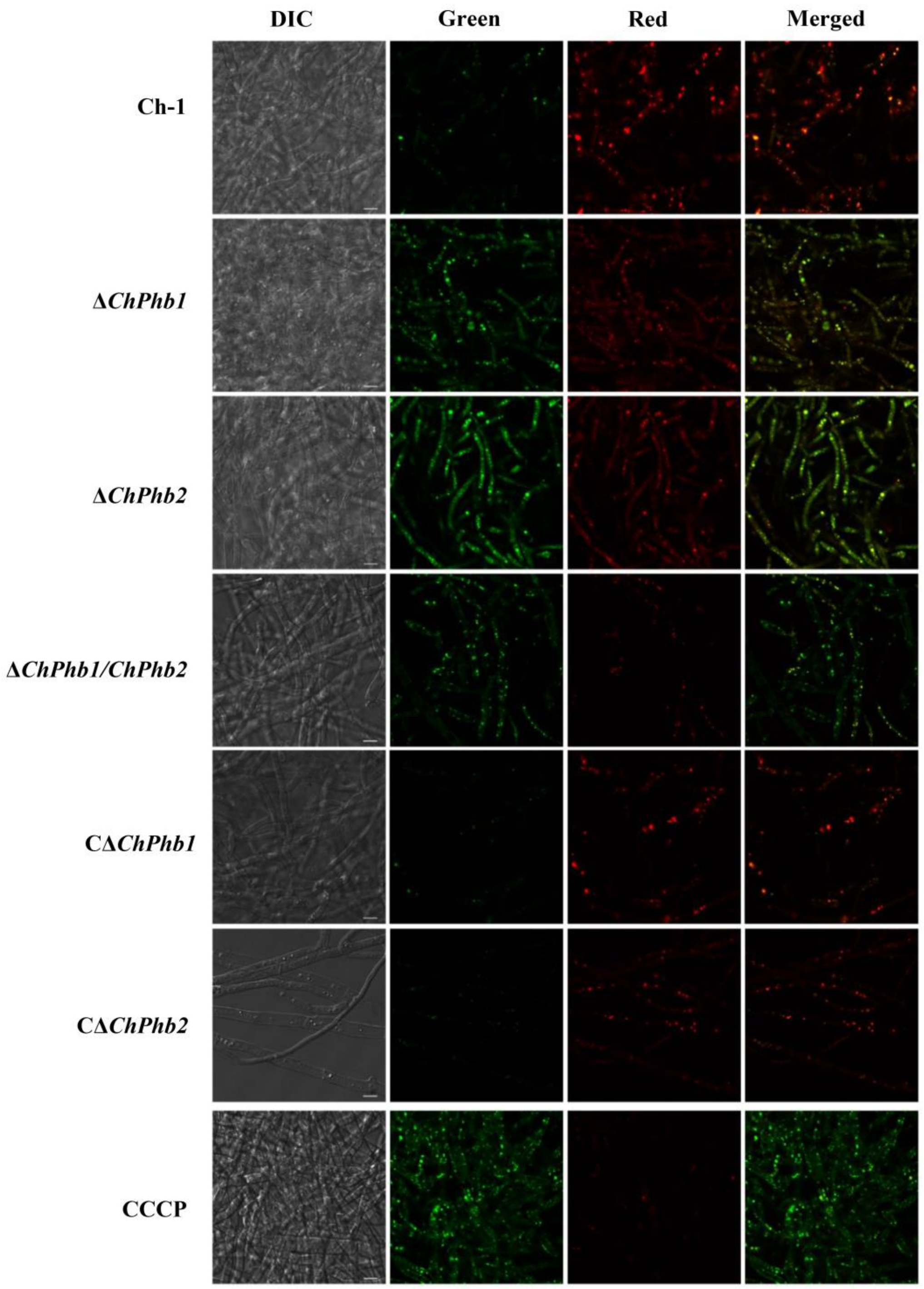

Supplementary Fig. 3. Fluorescent observation of ChPhb1, ChPhb2, and ChPhb1/ChPhb2 deletion and complementation mutants using JC-1 dye in hyphae of Ch-1 and various mutants. Scale bar, 5 μm.

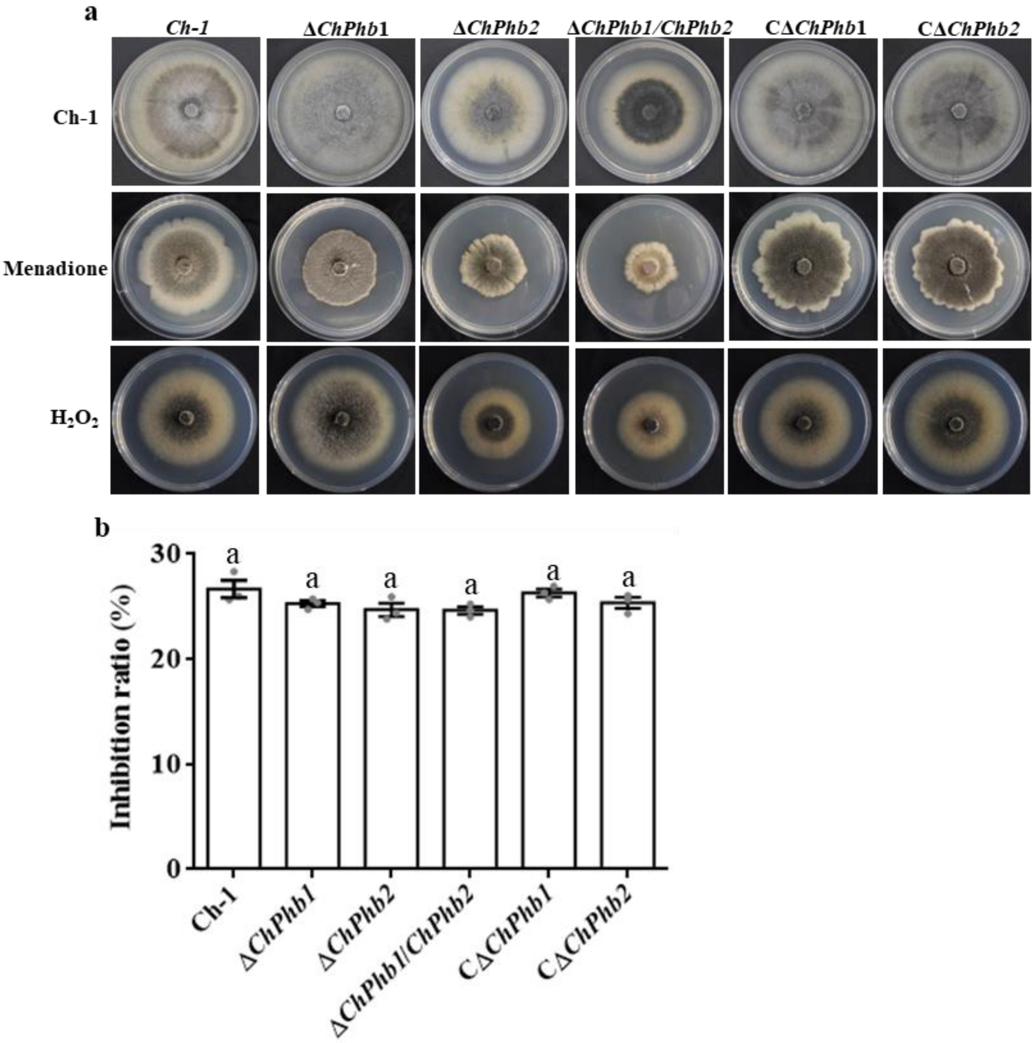

Supplementary Fig. 4. *ChPhb1* and *ChPhb2* were required for responses to mitochondrial ROS. a, Morphology of colonies of each strain subjected to ROS stress for 7 d at 25°C. **b**, Inhibition ratio calculated based on colony diameter of strains subjected to H_2O_2 for 7 d.

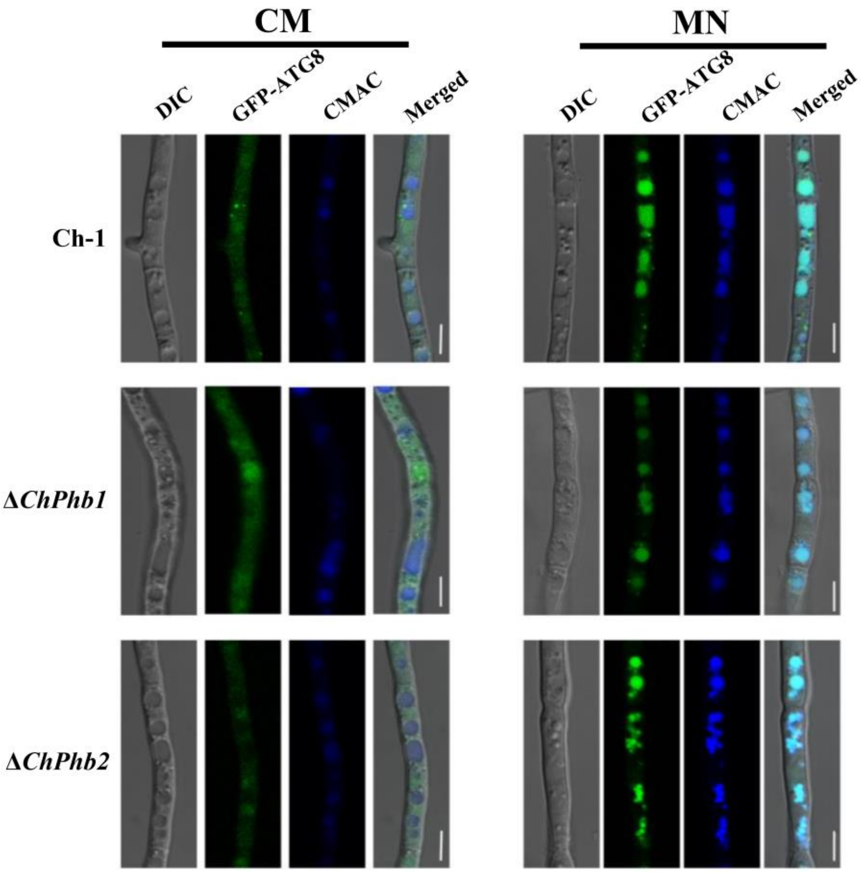

Supplementary Fig. 5. ChPhb1 and ChPhb2 are not required for non-specific autophagy. MN-starved mycelia were co-stained with 10 μM CMAC. Confocal microscopy of ATG8-GFP and CMAC-stained vacuoles was performed to detect autophagy in each strain. Scale bar, 5 μm.

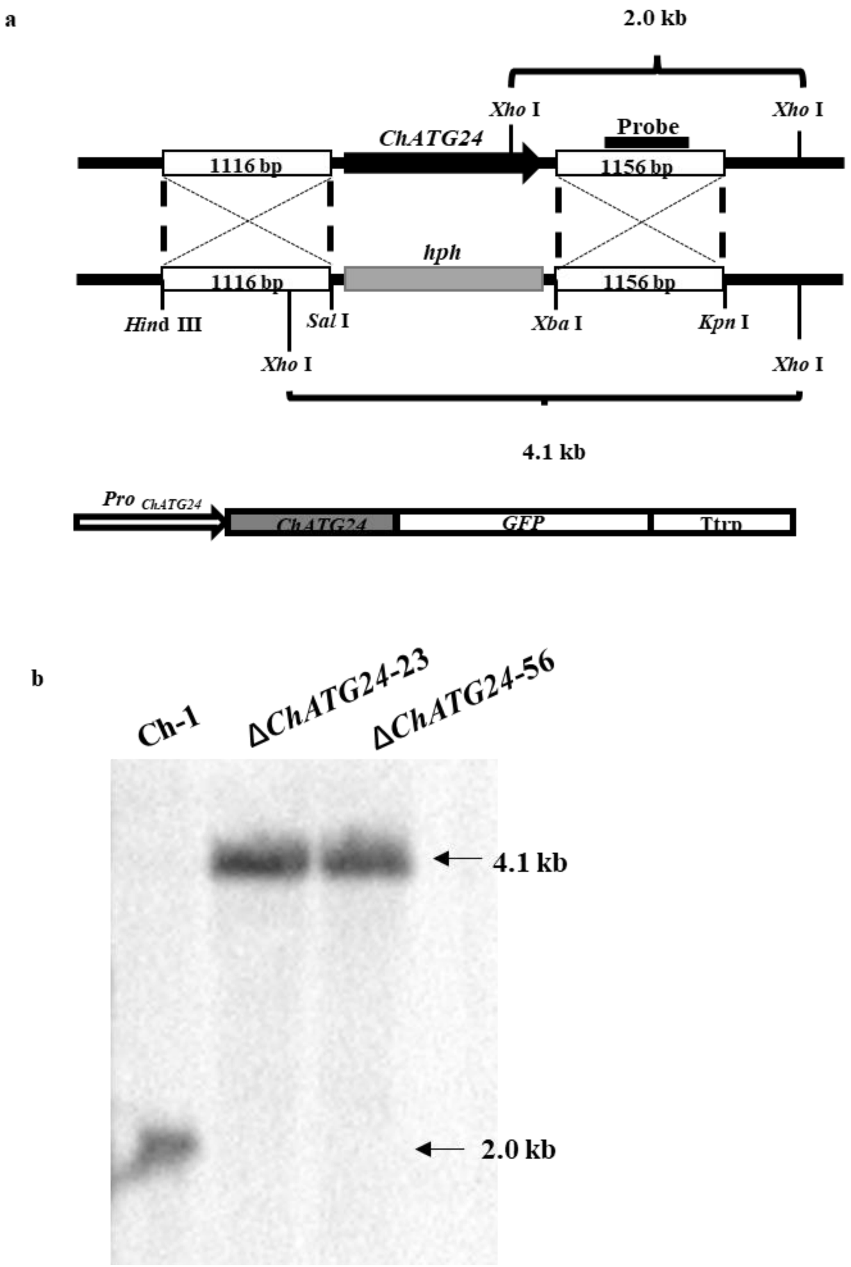

Supplementary Fig. 6. Characterization, targeted deletion, and complementation of *ChATG24*. **a**, Strategic map of the *ChATG24* deletion and complementation constructs and restriction enzyme sites. **b**, Southern blot confirmation of the *ChATG24* deletion mutants.

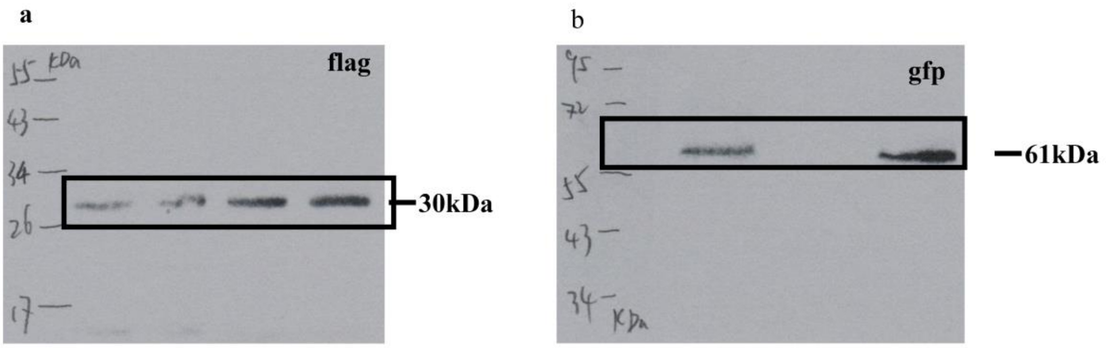

Supplementary Fig. 7. Western blot analysis of the interactions between ChPhb1 and ChPhb2. Box with dark line indicates results shown in Fig. 3b**.**

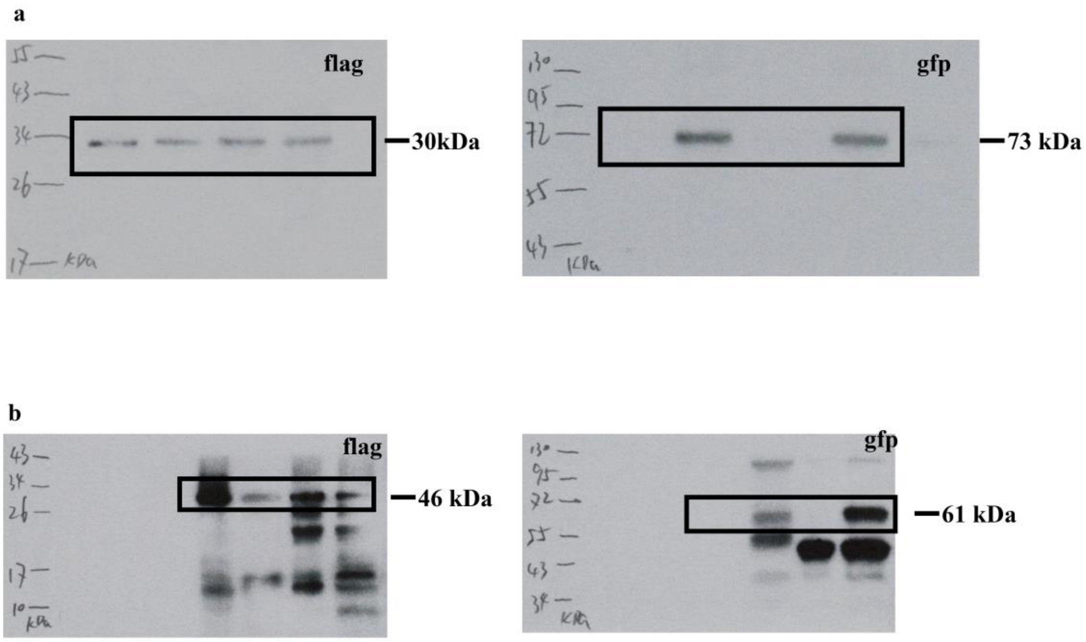

Supplementary Fig. 8. Western blot analysis of the interactions between ChPhb1 and ChATG24, ChPhb2 and ChATG24. a, Co-IP assays of the interactions between ChATG24 and ChPhb1. The lanes are explained in Supplementary Fig. 6b. **b**, Co-IP assays of the interactions between ChATG24 and ChPhb2. Box with dark line indicates results shown in Fig. 6c.

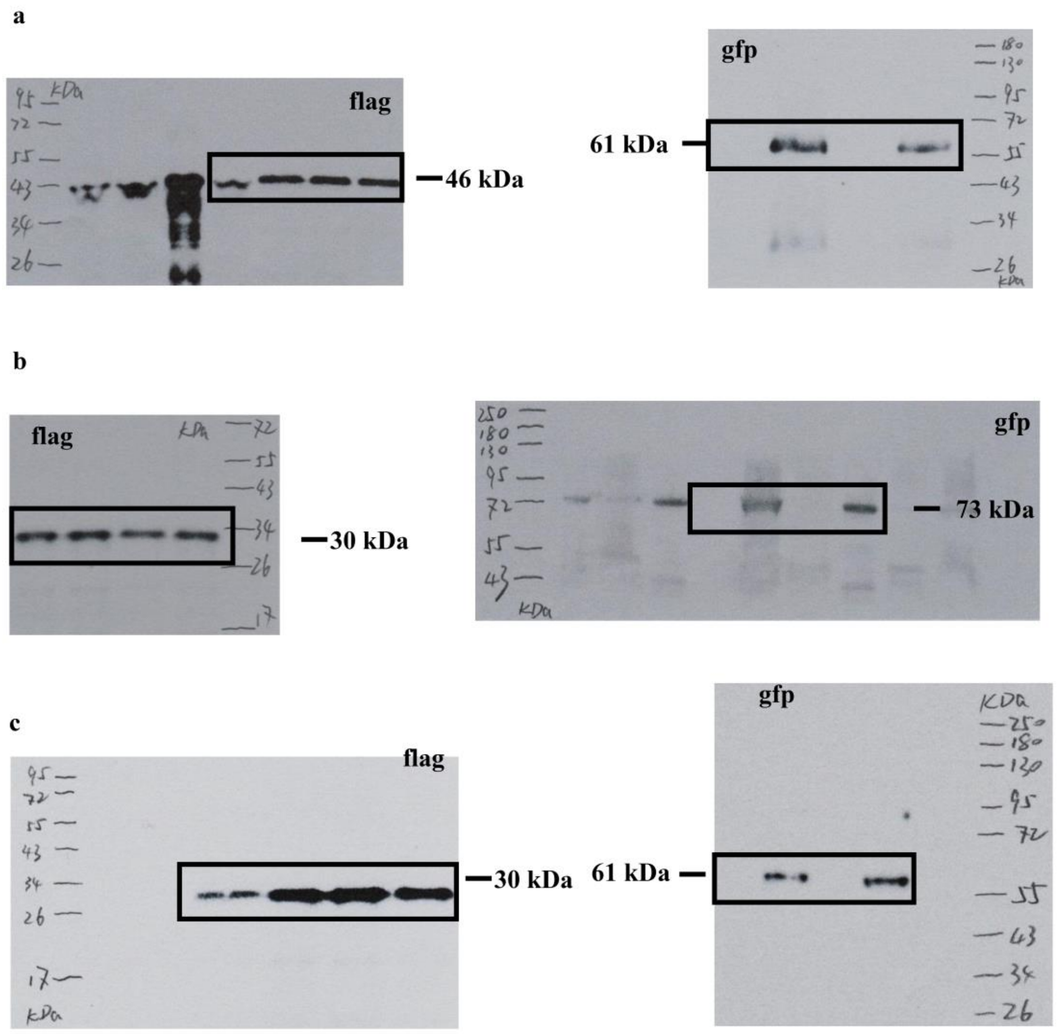

Supplementary Fig. 9. Western blot analysis of the interactions between ChPhb1 and ChPhb2, ChPhb1 and ChAtg24, or ChPhb2 and ChAtg24 in the absence of *ChATG24, ChPhb2* or *ChPhb1*. a, Co-IP assays of the interactions between ChATG24 and ChPhb2 in Δ*ChPhb1* mutant. **b**, Co-IP assays of the interactions between ChATG24 and ChPhb1 in Δ*ChPhb2* mutant. **c**, Co-IP assays of the interactions between ChPhb1 and ChPhb2 in Δ*ChATG24* mutant. Box with dark line indicates results shown in Fig. 6d.

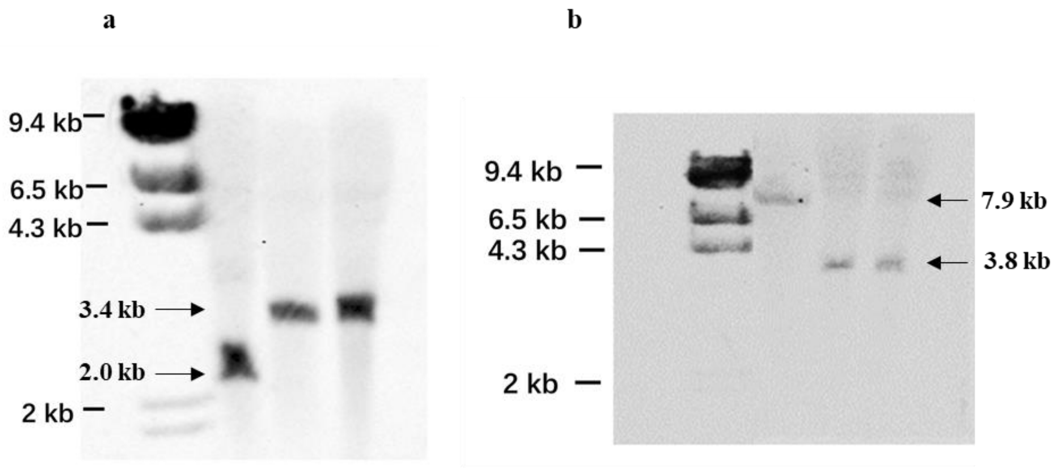

Supplementary Fig. 10. Southern blot confirmation of the *ChPhb1* and *ChPhb2* deletion mutants. a. The southern blot of *ChPhb1* deletion mutants. **b**, Southern blot confirmation of the ChPhb2 deletion mutants. The lanes are explained in Supplementary Fig. 1f.

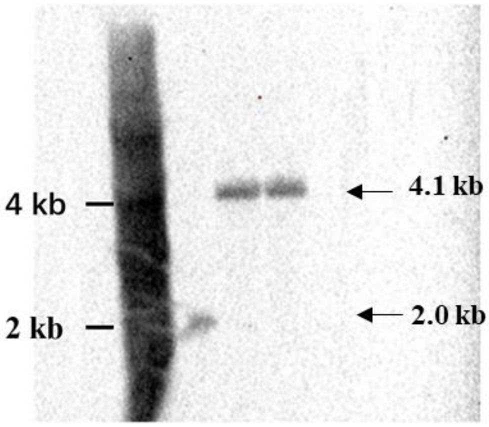

Supplementary Fig. 11. Southern blot confirmation of the *ChATG24* deletion mutants. The lanes are explained in Supplementary Fig. 6b.
