# Supplementary material for: Mitochondrial prohibitin complex regulates fungal virulence via ATG24-assisted mitophagy
Source: Commun Biol. 2022 Jul 14;5:698. doi: 10.1038/s42003-022-03666-5 (PMC9283515; doi:10.1038/s42003-022-03666-5)
Supplement: Supplementary file 3 — Description of Additional Supplementary Files [file 42003_2022_3666_MOESM3_ESM.pdf]

Description of Additional Supplementary Files

File name: Supplementary Data 1
Description: The source data behind the graphs in the paper.

File name: Supplementary Data 2
Description: Primers used in this study.
