# Supplementary material for: Mitochondrial prohibitin complex regulates fungal virulence via ATG24-assisted mitophagy
Source: Commun Biol. 2022 Jul 14;5:698. doi: 10.1038/s42003-022-03666-5 (PMC9283515; doi:10.1038/s42003-022-03666-5)
Supplement: Supplementary file 6 — Reporting summary [file 42003_2022_3666_MOESM6_ESM.pdf]

nature portfolio

Corresponding author(s): Lu Zheng
Last updated by author(s): Jun 6, 2022

Reporting Summary

Nature Portfolio wishes to improve the reproducibility of the work that we publish. This form provides structure for consistency and transparency in reporting. For further information on Nature Portfolio policies, see our Editorial Policies and the Editorial Policy Checklist.

Statistics

For all statistical analyses, confirm that the following items are present in the figure legend, table legend, main text, or Methods section.

n/a	Confirmed	
☐	☒	The exact sample size (n) for each experimental group/condition, given as a discrete number and unit of measurement
☐	☒	A statement on whether measurements were taken from distinct samples or whether the same sample was measured repeatedly
☐	☒	The statistical test(s) used AND whether they are one- or two-sided<br>*Only common tests should be described solely by name; describe more complex techniques in the Methods section.*
☒	☐	A description of all covariates tested
☒	☐	A description of any assumptions or corrections, such as tests of normality and adjustment for multiple comparisons
☐	☒	A full description of the statistical parameters including central tendency (e.g. means) or other basic estimates (e.g. regression coefficient) AND variation (e.g. standard deviation) or associated estimates of uncertainty (e.g. confidence intervals)
☒	☐	For null hypothesis testing, the test statistic (e.g. F, t, r) with confidence intervals, effect sizes, degrees of freedom and P value noted<br>*Give P values as exact values whenever suitable.*
☒	☐	For Bayesian analysis, information on the choice of priors and Markov chain Monte Carlo settings
☒	☐	For hierarchical and complex designs, identification of the appropriate level for tests and full reporting of outcomes
☒	☐	Estimates of effect sizes (e.g. Cohen's d, Pearson's r), indicating how they were calculated

Our web collection on statistics for biologists contains articles on many of the points above.

Software and code

Policy information about availability of computer code

Data collection	1.The full-length sequences of ChPhb1 and ChPhb2 were downloaded from the genomic database (http://fungi.ensembl.org/Colletotrichum_higginsianum/Info/Index) of C. higginsianum isolate IMI349063.<br>2. A phylogenetic tree was generated with MEGA software (version 7.0, http://www.megasoftware.net/index.php).<br>3.Data were subjected to analyses of variance (ANOVA) using SPSS 13.0 software (SPSS, Chicago, IL, USA).<br>4.qRT-PCR was conducted on a CFX96™ Real Time PCR system (Bio-Rad, Hercules, CA, USA).
Data analysis	1. Upon detecting a statistically significant treatment effect, means were separated using the test of least significant difference (LSD) test (P=0.05).<br>2.A phylogenetic tree was generated with MEGA software using the neighbor-joining (NJ) algorithm.<br>3.Immunoblot signals were measured using the ChemiDoc XRS+ system (Bio-Rad).

For manuscripts utilizing custom algorithms or software that are central to the research but not yet described in published literature, software must be made available to editors and reviewers. We strongly encourage code deposition in a community repository (e.g. GitHub). See the Nature Portfolio guidelines for submitting code & software for further information.

Data

Policy information about availability of data

All manuscripts must include a data availability statement. This statement should provide the following information, where applicable:

- Accession codes, unique identifiers, or web links for publicly available datasets
- A description of any restrictions on data availability
- For clinical datasets or third party data, please ensure that the statement adheres to our policy

All data relevant to this study are available from the authors upon reasonable request.

Field-specific reporting

Please select the one below that is the best fit for your research. If you are not sure, read the appropriate sections before making your selection.

☒ Life sciences ☐ Behavioural & social sciences ☐ Ecological, evolutionary & environmental sciences

For a reference copy of the document with all sections, see nature.com/documents/nr-reporting-summary-flat.pdf

Life sciences study design

All studies must disclose on these points even when the disclosure is negative.

Sample size	To understand the gene function of prohibitins and autophagy protein ChATG24 in Colletotrichum higginsianum, we generated the deletion mutants, and two random strains of each knockout mutant was confirmed by RT-PCR and Southern blotting. Only similar phenotypes between the two mutants was accepted. Then the mutants were selected for subsequent analyses.
Data exclusions	No data were excluded from the analyse.
Replication	In this study, each test was repeated three times. There were no significant differences between replicates.
Randomization	This is not relevant to our study. Our research focuses on the gene function of plant pathogenic fungi. The control group and the experiment groups were wild-type strain and knockout mutant strains, respectively.
Blinding	The blinding was not relevant to our study. The research object in our study is plant pathogenic fungi.

Reporting for specific materials, systems and methods

We require information from authors about some types of materials, experimental systems and methods used in many studies. Here, indicate whether each material, system or method listed is relevant to your study. If you are not sure if a list item applies to your research, read the appropriate section before selecting a response.

Materials & experimental systems

n/a	Involved in the study	
☐	☒	Antibodies
☒	☐	Eukaryotic cell lines
☒	☐	Palaeontology and archaeology
☒	☐	Animals and other organisms
☒	☐	Human research participants
☒	☐	Clinical data
☒	☐	Dual use research of concern

Methods

n/a	Involved in the study	
☒	☐	ChIP-seq
☒	☐	Flow cytometry
☒	☐	MRI-based neuroimaging

Antibodies

Antibodies used	Anti-Flag M2 affinity resins (Sigma Aldrich, USA, M8823), anti-FLAG (Sigma Aldrich, USA, A8592) and anti-GFP (TransGen Biotech, China,HT801-01) antibodies were used in this study.
Validation	1. Anti-flag M2 affinity resins are 4% agarose beads that can bind to anti-Flag M2 (mouse monoclonal) antibodies.<br>2. The M2 antibody recognizes the N-terminal, N-terminal and C-terminal of FLAG sequences. This can realize the detection and capture of fusion proteins containing FLAG peptide sequences.<br>3. The GFP antibody is highly specific to the C-terminal or N-terminal GFP tags of recombinant proteins and is suitable for qualitative or quantitative detection of GFP fusion expressed proteins.
